# Novel Divergent Polar Bear-Associated Mastadenovirus Recovered from a Deceased Juvenile Polar Bear

**DOI:** 10.1128/mSphere.00171-18

**Published:** 2018-07-25

**Authors:** Anisha Dayaram, Kyriakos Tsangaras, Selvaraj Pavulraj, Walid Azab, Nicole Groenke, Gudrun Wibbelt, Florian Sicks, Nikolaus Osterrieder, Alex D. Greenwood

**Affiliations:** a Leibniz Institute for Zoo and Wildlife Research, Berlin, Germany; b Department of Translational Genetics, The Cyprus Institute of Neurology and Genetics, Nicosia, Cyprus; c Institut für Virologie, Zentrum für Infektionsmedizin, Freie Universität Berlin, Berlin, Germany; d Tierpark Berlin, Berlin, Germany; e Department of Veterinary Medicine, Freie Universität Berlin, Berlin, Germany; University of Zurich

**Keywords:** adeno-associated virus, evolutionary biology, novel virus, phylogenetic analysis, polar bear

## Abstract

Polar bears in captivity can be exposed to opportunistic pathogens not present in their natural environments. A 4-month-old polar bear (Ursus maritimus) living in an isolated enclosure with his mother in the Tierpark Berlin, Berlin, Germany, was suffering from severe abdominal pain, mild diarrhea, and loss of appetite and died in early 2017. Histopathology revealed severe hepatic degeneration and necrosis without evidence of inflammation or inclusion bodies, although a viral infection had been suspected on the basis of the clinical signs. We searched for nucleic acids of pathogens by shotgun high-throughput sequencing (HTS) from genomic DNA and cDNA extracted from tissue and blood. We identified a novel *Mastadenovirus* and assembled a nearly complete genome from the shotgun sequences. Quantitative PCR (qPCR) revealed that viral DNA was present in various concentrations in all tissues examined and that the highest concentrations were found in blood. Viral culture did not yield cytopathic effects, but qPCR suggested that virus replication was sustained for up to three passages. Positive immunofluorescence staining confirmed that the virus was able to replicate in the cells during early passage. Phylogenetic analysis demonstrated that the virus is highly divergent compared to other previously identified *Mastadenovirus* members and basal to most known viral clades. The virus was found only in the 4-month-old bear and not in other captive polar bears tested. We surmised, therefore, that the polar bear was infected from an unknown reservoir, illustrating that adenoviral diversity remains underestimated and that cross-species transmission of viruses can occur even under conditions of relative isolation.

**IMPORTANCE** Cross-species transmission of viral pathogens is becoming an increasing problem for captive-animal facilities. This study highlights how animals in captivity are vulnerable to novel opportunistic pathogens, many of which do not result in straightforward diagnosis from symptoms and histopathology. In this study, a novel pathogen was suspected to have contributed to the death of a juvenile polar bear. HTS techniques were employed, and a novel *Mastadenovirus* was isolated. The virus was present in both the tissue and blood samples. Phylogenetic analysis of the virus at both the gene and genome levels revealed that it is highly divergent to other known mastadenoviruses. Overall, this study shows that animals in isolated conditions still come into contact with novel pathogens, and for many of these pathogens, the host reservoir and mode of transmission are yet to be determined.

## Abstract (precis)

Cross-species transmission of viral pathogens is becoming an increasing problem for captive-animal facilities. This study highlights how animals in captivity are vulnerable to novel opportunistic pathogens, many of which do not result in straightforward diagnosis from symptoms and histopathology. In this study, a novel pathogen was suspected to have contributed to the death of a juvenile polar bear. HTS techniques were employed, and a novel *Mastadenovirus* was isolated. The virus was present in both the tissue and blood samples. Phylogenetic analysis of the virus at both the gene and genome levels revealed that it is highly divergent to other known mastadenoviruses. Overall, this study shows that animals in isolated conditions still come into contact with novel pathogens, and for many of these pathogens, the host reservoir and mode of transmission are yet to be determined.

## INTRODUCTION

Captive polar bears are vulnerable to opportunistic pathogens present in their anthropogenic environments. Captive-animal environments may inadvertently serve as hot spots for pathogens to concentrate and mix in nonsympatric species. This has led to polar bears being exposed to pathogens they would never encounter in their natural environment (1–4). Viral pathogens such as equine herpesvirus (EHV) types 1 (1) and 9 (3, 5), West Nile virus (WNV) (2), and suid herpesvirus (SuHV) (4) have been responsible for many serious and often lethal infections in captive polar bears in Europe and the United States. Polar bears at the Zoological Gardens, Wuppertal, Germany, were found to be infected with EHV-1, while polar bears at the Zoological Society of San Diego were infected with EHV-9. However, in both cases, the polar bears were not in direct contact with any equine species, and the source and mode of transmission have remained undetermined. This suggests that there may be an indirect vector(s) enabling the transmission of many viruses infecting different animals in zoos that never come into direct physical contact.

In March 2017, a 4-month-old polar bear (Ursus maritimus) named Fritz developed a sudden onset of mild diarrhea and loss of appetite in the Tierpark Berlin, Berlin, Germany. Three days later, he died with severe abdominal pain. At necropsy, a severely enlarged liver (hepatomegaly) was detected. Histopathology revealed severe hepatic degeneration and necrosis. There was no evidence of inflammation or intranuclear or cytoplasmic inclusion bodies. A search for viral particles using transmission electron microscopy (TEM) in negative-stained homogenates from fresh liver tissue and intestinal mucosa was negative. Likewise, TEM investigations of several epoxy-embedded liver samples did not detect intranuclear or intracellular viral particles. We performed an in-depth investigation at the molecular level to identify potential pathogens that could be responsible for the disease. We used high-throughput sequencing techniques (HTS) and classical virological methodology to examine tissue from liver, kidney, prescapular lymph nodes, large intestine, and blood. We identified and characterized a nearly complete genome of a novel *Mastadenovirus* that was present in all tissue and blood samples tested and is the first to be recovered from the Ursidae family.

Mastadenoviruses belong to the family *Adenoviridae* and have linear double-stranded DNA genomes that range in size from 26 to 43 kb (6). They infect a broad range of host vertebrates and are known to be extremely stable in the environment, suggesting that they may have multiple transmission routes (7, 8). Most adenoviruses have been shown to have restricted host specificity (9) and usually cause clinical signs that include enteritis and respiratory disease, but other clinical signs can also result from infection (10–12, 53). Adenoviruses are used as gene therapy vectors to treat genetic disorders and cancer (13). They are important vectors, as they can accommodate large pieces of foreign DNA and target multiple tissues. Additionally, animal adenoviruses have been successfully engineered as vectors for gene delivery in humans (14).

To date, no adenoviruses have been isolated from polar bears or any other species of bear. However, in 1948, the first probable occurrence of an adenovirus infecting a polar bear was reported but was never confirmed (15). In addition, an adenovirus was isolated from two black bear cubs (Ursus americanus) that died within 1 to 4 days after they exhibited signs that included salivation, emesis, convulsions, periodic nystagmus, and paddling of the legs (16). In this case, a polar bear also kept in the same facility and suffering from an unknown condition recovered after administration of canine hepatitis (hepatitis contagiosa canis [HCC]) antiserum. In this case study, the necropsy findings of the black bear cubs were confirmed by both histopathology and electron microscopy, results that led to HCC diagnosis.

The viral genome determined was divergent from all known adenoviruses, and genetic distances for most genes of the new virus (polar bear-associated mastadenovirus 1 [PbAdV-1]) were equidistant from known viruses in GenBank. Phylogenetic analysis of the viral genome and genes failed to indicate the reservoir, and analysis suggests that it is a mammalian *Mastadenovirus* basal to most other adenoviruses. In addition, the virus grew on two cell lines, but it was unclear whether it was noncytopathic to the cell lines or whether they simply did not fully support effective replication of the virus. The results suggest that like other pathogens, even in isolated facilities such as the quarantined enclosure in which the baby polar bear was kept with its mother, cross-species transmission of viruses can occur in captivity.

## RESULTS

### Pathology and TEM.

The only significant histopathological finding was an acute severe hepatic degeneration and necrosis associated with marked hemorrhage. There was no evidence of an inflammatory reaction or of intranuclear or intracytoplasmic inclusion bodies in any of the internal organs. Additional special stains did not reveal any evidence of the presence of another pathogen(s). The intestine, kidneys, heart, and brain had no morphological lesions. The lungs had damage related to reanimation attempts, and the spleen and lymph nodes showed age-related apoptosis of lymphoid cells and reactive germinal centers in the lymphoid follicles.

### Novel adenovirus genome characterization and phylogenetic analysis.

**FIG 1:**
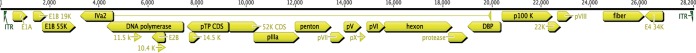
Genome organization of PbAdV-1. The nearly complete 28,200-bp genome assembled from the Illumina sequencing reads is shown in black. ORFs encoding genes are shown in yellow along with their orientation in the genome. 19K, 19,000; CDS, coding sequence; DBP, DNA-binding protein; ITR, inverted terminal repeat.

Using standard homology-based searches at a 95% similarity threshold, no virus-specific sequences could be identified in any of the samples beyond known polar bear endogenous retroviruses. Using a bioinformatic pipeline specifically developed for detection of divergent viral sequences from HTS data (Viral Identification Pipeline [VIP]), a novel adenovirus with a nearly complete ~28-kb linear genome was assembled from Illumina paired-end reads, which we named polar bear-associated adenovirus 1 (PbAdV-1) (Fig. 1) (GenBank accession no. MH115806). Illumina reads aligning to the genome were present in all tissue samples that were sequenced, including liver, prescapular lymph nodes, large intestine, and kidney. Major open reading frames (ORFs) encompassing all genes, including the *hexon* and *pol* genes, were identified (Fig. 1). Although the genes were quite divergent with a pairwise identity of ~40%, the viral gene order was typical of adenoviruses. Contamination in HTS has proved troublesome in the past (17). In order to rule out potential contamination from reagents, including DNA extraction kits and library building reagents, we used negative controls in addition to showing that the virus was able to be directly amplified by PCR from the blood of the polar bear using both *pol*- and *hexon*-specific primer sets.

**FIG 2:**
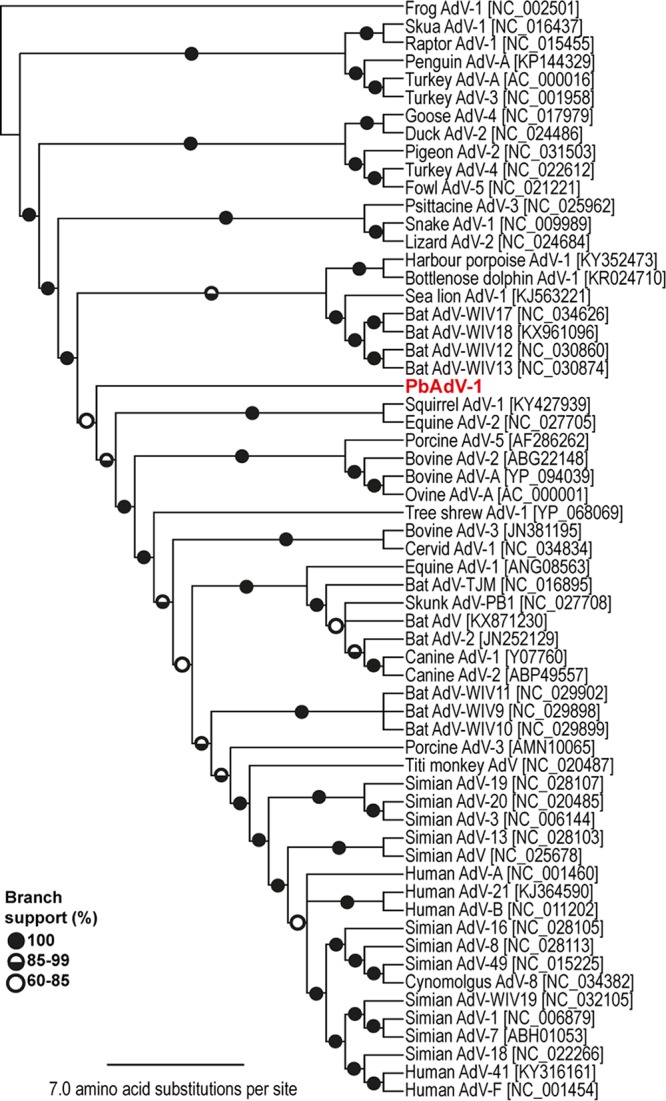
RAxML maximum likelihood phylogenetic tree of all identified genes from adenoviruses using LG gamma substitution model with 20 maximum likelihood searches and 500 rapid bootstrap replicates and the frog adenovirus 1 (NC_002501.1) as an outgroup. PbAdV-1 is shown in red.

Phylogenetic analysis of both individual genes and the full genome did not fully resolve the evolutionary relationship to other adenoviruses, indicating that PbAdV-1 is very divergent (Fig. 2; see Fig. S1 in the supplemental material). The *hexon* gene encodes a major virus capsid protein in adenoviruses (18) and was 2,749 bp in length in PbAdV-1. Phylogenetic analysis of this gene shows it in a nonbasal group and distantly related to other *hexon* genes from other known adenoviruses (Fig. S1) but more closely related to the genes of viruses isolated from mammals. This same result was observed in the phylogenetic analysis of the DNA polymerase gene (*pol*). The *pol* gene in adenoviruses is conserved, as it is responsible for replication of the viral genome (19). In PbAdV-1, the *pol* gene was 3,280 bp, again phylogenetic analysis indicated that the gene was distantly related to other *pol* genes from adenoviruses currently known to infect mammals (Fig. S1). Combining genes from PbAdV-1 placed the virus in a basal position to a clade containing several mammalian orders, although the most basal adenoviral clade included two cetacean adenoviruses, four bat adenoviruses, and one phocid adenovirus (Fig. 2 and Table S1).

10.1128/mSphere.00171-18.1 FIG S1  RAxML maximum likelihood phylogenetic trees of major genes from adenoviruses using the LG gamma substitution model with 20 maximum likelihood searches and 500 rapid bootstrap replicates and the frog adenovirus 1 (NC_002501.1) as an outgroup. The polar bear adenovirus genes are shown in red. (A) *Hexon* gene; (B) *pol* gene. Download FIG S1, PDF file, 0.2 MB. Copyright © 2018 Dayaram et al. 2018 Dayaram et al. This content is distributed under the terms of the Creative Commons Attribution 4.0 International license.

10.1128/mSphere.00171-18.3 TABLE S1  Primers designed for the *hexon* and *pol* genes to confirm putative adenovirus PbAdV-1. Download TABLE S1, DOCX file, 0.01 MB. Copyright © 2018 Dayaram et al. 2018 Dayaram et al. This content is distributed under the terms of the Creative Commons Attribution 4.0 International license.

### Virus detection by PCR and qPCR.

All tissue samples (liver, kidney, prescapular lymph nodes, and large intestine) and blood samples from polar bear Fritz were screened using three specific primers targeting both the *hexon* and *pol* genes of PbAdV-1. All samples gave positive results with all primer sets (Table 1). In addition, quantitative PCR (qPCR) was carried out to quantify the *pol* gene in PbAdV-1 from viral DNA in both tissue and blood samples. The results show that blood samples had the lowest threshold cycle (*C*_*T*_) value, indicating that it had the largest amount of viral DNA (*C*_*T*_= 19.84), followed by the prescapular lymph nodes (*C*_*T*_ = 20.26) and the liver (*C*_*T*_ = 20.98). The large intestine had the smallest amount of novel adenovirus DNA present (*C*_*T*_ = 25.16). PCR of both the *pol* and *hexon* genes was performed on other polar bear samples to identify other potential hosts. Blood and liver samples from eight captive polar bears from five European zoos were screened for the virus. The PCR results show that all samples screened apart from Fritz were negative for PbAdV-1 (Table S2).

10.1128/mSphere.00171-18.4 TABLE S2  PCR results for the screening of other polar bear samples from around Europe for the PbAdV-1 targeting the *hexon* and *pol* genes. Download TABLE S2, DOCX file, 0.01 MB. Copyright © 2018 Dayaram et al. 2018 Dayaram et al. This content is distributed under the terms of the Creative Commons Attribution 4.0 International license.

**TABLE 1:** PCR and qPCR performed on the DNA extracted from blood and tissue samples from polar bear Fritz

Sample screened	Detection by PCR using the following primer^a^:						Mean *C*_*T*_ value^b^ detected using SYBR green qPCR
	Hexon 1	Hexon 2	Hexon 3	pol 1	pol 2	pol 3	
Blood	+	+	+	+	+	+	19.84
Liver	+	+	+	+	+	+	20.98
Kidney	+	+	+	+	+	+	23.25
Prescapular lymph node	+	+	+	+	+	+	20.26
Large intestine	+	+	+	+	+	+	25.16

^a^ The PCR targeted the *hexon* and *pol* genes using the primers listed in Table 1. SYBR green-based comparative qPCR of the *pol* gene for PbAdV-1 was performed on DNA extracted from blood, liver, kidney, prescapular lymph nodes, and large intestine. Symbol: +, a positive result from PCR.

^b^ The *C*_*T*_ shows the mean of each sample that was run in triplicate.

### Polar bear *CXADR* gene analysis.

The coxsackievirus and adenovirus receptor (CAR) is a protein encoded by the *CXADR* gene and is the cellular receptor in the host that allows adenovirus cellular entry. The *CXADR* gene was examined to see if any mutations were similar to *CXADR* genes in other host reservoirs for mastadenoviruses or if there were any unusual features of the polar bear receptor that might suggest why the bear was infected. The *CXADR* gene was extracted from the polar bear genome (SRA092289) and used as the reference to align the whole-genome data obtained from polar bear Fritz. The resulting alignment had a high coverage on the entire region of the gene (33,293 bp). Nucleotide percentage pairwise identity analysis of the polar bear *CXADR* to other mammals using BLAST shows that the gene is most closely related to the giant panda *CXADR* gene with 83.5% nucleotide identity (Table S3). The Fritz *CXADR* gene consensus sequence was examined for unique polymorphisms. The analysis identified a limited number of observed polymorphisms, including one 16-bp deletion indel (16274 to 16289 bp), that were not present in other *CXADR* genes from other mastadenovirus hosts.

10.1128/mSphere.00171-18.5 TABLE S3  GenBank accession numbers and species origin for adenovirus sequences used in the MAFFT v7.017 alignment to construct the RAxML maximum likelihood phylogenetic trees for the *pol* and *hexon* genes and full genome. Download TABLE S3, DOCX file, 0.02 MB. Copyright © 2018 Dayaram et al. 2018 Dayaram et al. This content is distributed under the terms of the Creative Commons Attribution 4.0 International license.

### Cell culture.

**FIG 3:**
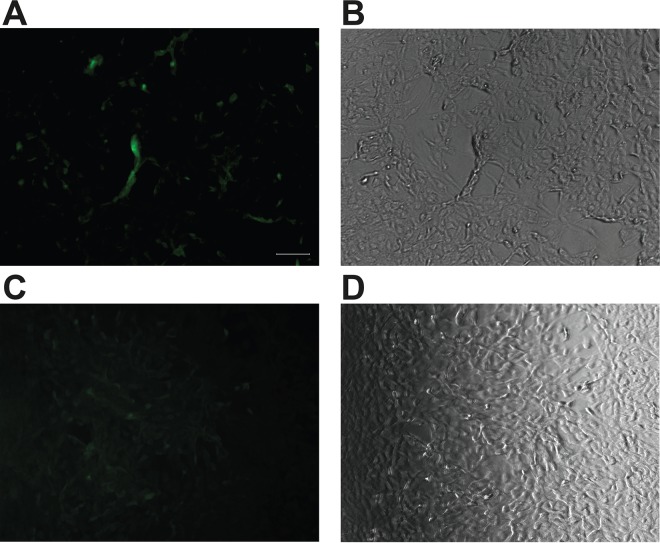
Indirect immunofluorescence (IF) staining 48 h postinoculation for adenoviral antigens in CrFK cells infected with supernatant P1 from the prescapular lymph node. IF staining carried out using goat adenovirus antibody diluted 1:20 in 3% BSA in PBS as the primary antibody and anti-goat FITC diluted 1:100 in 3% BSA in PBS as the secondary antibody. (A) Bright green positive IF staining of cytoplasm of adenovirus-infected CrFK cells. Bar, 100 μm. (B) Corresponding bright-field image of panel A. (C) An uninfected CrFK cell control shows no IF signal. (D) Corresponding bright-field image of panel C. Scale bar is 100 μm.

In an effort to determine virus viability and potential hosts, cell culture was conducted using liver, kidney, and lymph node tissue samples on 11 different cell lines and passaged consecutively three times. Culture of blood-derived virus could not be performed, as the sample was severely hemolyzed. Cells were observed for cytopathic effect (CPE) of the virus, and qPCR analysis was performed to assess infectivity of PbAdV-1. The results from the initial cell culture showed that infection occurred in three cell lines, Madin-Darby canine kidney II (MDCK II), Vero, and Crandell Rees feline kidney (CrFK). Immunofluorescence microscopy was used to confirm PbAdV-1 infection of CrFK, MDCK II, and Vero cells. The positive immunofluorescence (IF) signal of the CrFK cell lines demonstrate that 48 h postinoculation, the cells are infected by the virus and are able to replicate in the cell line (Fig. 3). However, although the virus infects the cells, microscopy also shows that there is no clear CPE in any cell line (Fig. 3, Fig. S2, and Table 2). The IF signal in both MDCK II and Vero cell lines was extremely weak (data not shown); at 48 hours postinoculation, there was minimal infection of the cells by the virus and no visible CPE.

10.1128/mSphere.00171-18.2 FIG S2  Indirect immunofluorescence (IF) staining 48 h postinoculation for adenoviral antigens in CrFK cells infected with supernatant P1 from the prescapular lymph node. IF staining was conducted using a primary antibody (goat adenovirus antibody diluted 1:20 in 3% BSA in PBS) and secondary antibody (anti-goat FITC diluted 1:100 in 3% BSA in PBS). For the positive samples, panels A, C, E, G, and I show bright green positive IF staining of cytoplasm of adenovirus-infected CrFK cells, while panels B, D, F, H, and J show the corresponding bright-field images for positive samples. For the negative-control samples, panels A, C, E, G, and I depict uninfected CrFK cell controls that show no IF signal, while panels B, D, F, H, and J show the corresponding bright-field images for negative samples. Scale bar is 100 μm. Download FIG S2, PDF file, 8 MB. Copyright © 2018 Dayaram et al. 2018 Dayaram et al. This content is distributed under the terms of the Creative Commons Attribution 4.0 International license.

**TABLE 2:** SYBR green-based comparative qPCR of *pol* gene for PbAdV-1^a^

Tissue	Cell line	*C*_*T*_for DNA from cells		
		Passage 1	Passage 2	Passage 3
Liver	CrFK	38	36.4	33
	MDCK II	−	−	−
	Vero	−	−	−
	BHK	−	−	−
Kidney	CrFK	−	−	30
	MDCK II	29	−	−
	Vero	−	−	−
	BHK	−	−	−
Lymph node	CrFK	26	28	31
	MDCK II	24	30	28
	Vero	−	29	31
	BHK	−	−	−

^a^ qPCR was performed on DNA extracted from cell culture of supernatant from liver, kidney, and lymph node tissue on the CrFK, MDCK II, Vero and BHK cell lines after 1, 2, and 3 passages. −, negative result.

Kidney and lymph node tissues were positive for the adenovirus *pol* gene by qPCR with *C*_*T*_ values of 20 and 17, respectively (Table 1). Liver tissue culture was negative for adenovirus. qPCR analysis of cell culture samples were performed for infected CrFK, MDCK II, Vero, and baby hamster kidney (BHK) cells. In passage 1 (P1), CrFK cells infected with lymph node and liver homogenates had *C*_*T*_ values of 24 and 38, respectively. In subsequent passages (P2 and P3), *C*_*T*_ values increased in CrFK cells with the lymph node inocula (P3, *C*_*T*_ = 31) (viral DNA quantity decreased), whereas slight decreases in *C*_*T*_ values were observed for liver tissue-infected CrFK cells (P3, *C*_*T*_ = 33) (viral DNA quantity increased). CrFK cells inoculated with kidney tissue homogenates had detectable *C*_*T*_ only at P3 (*C*_*T*_ = 30). Lymph node tissue-infected MDCK II cells had a *C*_*T*_ value of 24 and 28 at P1 and P3, respectively, whereas kidney tissue-infected MDCK II cells had detectable *C*_*T*_ only at P1 (*C*_*T*_ = 29), and adenoviral DNA could not be detected in subsequent passages. Further, only lymph node tissue-infected Vero cells had *C*_*T*_s of 29 and 31 at P2 and P3, respectively. BHK cells remained negative for viral DNA in all passages, in spite of inoculation with the same tissue inocula used for MDCK II, CrFK, and Vero cells. The viral DNA levels at P1 to P3 did not exceed the basal DNA levels present in the inocula.

## DISCUSSION

In this study, we have employed the use of HTS techniques coupled with traditional cell culture and microscopy to identify a novel adenovirus that we named PbAdV-1 from tissue and blood samples taken from a deceased captive polar bear. The pathology results showed acute severe noninflammatory liver damage. Lesions typical of adenoviral infections (20) were not observed in any of the major organs. Investigations by TEM also did not yield evidence for a virus infection. Only the results obtained from HTS for various tissues confirmed a possible novel adenovirus isolated in both tissue and blood samples.

Difficulties that were encountered in both cell culture isolation attempts and identifying PbAdV-1 using electron microscopy have proven troublesome for previous studies such as the identification of the bottlenose dolphin adenovirus (21). The virus was believed to have replicated to some extent in the HeLa cells; however, electron microscopy could not give conclusive results on identifying adenovirus particles in the infected cells similar to PbAdV-1. In addition to this, the bottlenose dolphin adenovirus did not show typical tissue tropism for liver and lungs, much like the PbAdV-1. This shows that culturing novel adenoviruses may prove difficult if little or nothing is known about the potential host and the tissue the virus replicates in. This is particularly complicated in cases like Fritz where it is highly likely that the polar bear was an accidental host and no established polar bear reagents, e.g., cell cultures, exist.

The results from the PCR of the *pol* and *hexon* genes coupled with the qPCR results indicate that the highest viral copy number was observed in the blood, although all organs were positive for the virus (Table 1). This suggests that the virus may replicate in blood cells and not in a specific organ, as no tissue lesions were observed. The presence of the virus in blood would explain why all organs tested were positive for the virus, as all organs are connected to the circulatory system. Unfortunately, blood was not immediately examined using microcopy or negative staining and by the time qPCR was carried out, the blood had been frozen and thawed multiple times. Viremia has been reported for other adenoviruses in pygmy marmosets (Callithrix pygmaea) (22), kowari (Dasyuroides byrnei) (23), sea lions (Zalophus californianus) (24), and reptiles (25). In these studies, adenoviral DNA was often isolated and amplified from several organs, even though the pathology did not always reflect a typical adenovirus infection, which is similar to what was observed with PbAdV-1.

The polar bear coxsackievirus and adenovirus receptor (CAR), which is widely expressed in many cell types, including hematopoietic cells, was also characterized. CAR is also expressed in several tissues, including heart, brain, epithelial, and endothelial cells (26, 27). In the polar bear Fritz, the gene shared close nucleotide pairwise identity with those of other members of the Ursidae family such as the giant panda; however, it is not known how the 16-bp deletion in the gene identified here would affect binding of adenoviruses, as it was not in a conserved receptor-binding site. Studies have demonstrated that the conserved CAR-binding sites on the fiber proteins such as the AB loop are crucial for successful virus attachment to the CAR-binding site, so mutations in these conserved regions of the gene are likely to be deleterious (28, 29). The lack of tropism in the tissues examined may also be explained by other factors linked to CAR such as virus replication being reduced in cells with downregulated CAR expression and the abundance and regulation of CAR that can influence the availability of the correct receptor (30). However, the extremely wide taxonomic distribution of mammalian hosts for related mastadenoviruses suggests that there is little if any restriction of these viruses to a specific mammalian taxon and that it was exposure to the virus rather than specific mutations in polar bear CAR or any other adenoviral receptor that resulted in Fritz’s infection.

The cell culture results in 11 different cell lines did not give any further indication as to potential hosts or cell type preference for PbAdV-1. The qPCR and immunofluorescence results from the cell culture indicate that the virus was able to infect MDCK II, Vero, and CrFK cells, but not BHK cells; however, replication was not efficient and not beyond the third passage. The lack of CPE in MDCK II, Vero, and CrFK cells suggests either that these cell lines do not support efficient replication of the virus or that the virus is noncytopathic in these cell lines. However, indirect immunofluorescence assay (IFA) staining within the cytoplasm of CrFK and Vero cells infected with the lymph node tissue homogenate showed that expression of viral antigens is indicative of at least some virus replication, although the signal was stronger in the CrFK cells. The cell culture results support the lack of histopathological evidence for viral infection, as neither inclusion bodies nor individual viral particles could be found in tissue sections (data not shown). Taken together, our results may suggest that the presence of the adenovirus in the polar bear was not the primary cause of its death. However, it is possible that the infection with PbAdV-1 could have compromised the immune system of the bear, leaving it susceptible to other pathogens or toxins. Fowl adenovirus 8 infections in chickens can for example, result in compromised immune responses in chicken (31).

The phylogenetic analysis of PbAdV-1 shows that it groups with other mastadenoviruses identified in mammals; however, it likely diverged from other known adenoviruses some time ago. Only the harbor porpoise AdV-1 was in a more basal position among mammalian adenoviruses. Neither individual genes nor the combined genomic sequence could resolve the phylogeny of PbAdV-1 with confidence (Fig. 2; see Fig. S1 in the supplemental material). The lack of similarity to other adenoviruses identified in animals provided no indication of potential hosts for the virus. The polar bear was 4 months old and was restricted in range within an indoor enclosure with its mother. The mother was fed beef. Phylogenetically, PbAdV-1 is not closely related to known domestic cattle adenoviruses, and therefore, a dietary route of infection is unlikely, particularly since the animal had not been weaned. Rodents and bats can enter most enclosures, and it is therefore conceivable that an as yet unidentified rodent or bat species transmitted the virus to Fritz. The mother was not sampled at the time of death, and it is unclear if she was infected as well though she has never exhibited symptoms of any kind. These results in conjunction with the negative PbAdV-l result for the other polar bears screened suggests that PbAdV-1 represents a new lineage (Table S2).

In summary, we report a novel adenovirus associated with the death of a 4-month-old polar bear. As the polar bear was not in direct contact with other animal species, the investigation of indirect viral vectors such as rodents, bats, water sources, and fomites may prove useful in giving further insights into pathogens the bear may have been indirectly exposed to. This highlights that there are numerous pathways where captive animals are able to come into contact with pathogens, and these need to be considered when trying to establish transmission routes and host specificity of novel viruses.

## MATERIALS AND METHODS

### Pathology.

The polar bear was received from the zoo five hours after death. Tissue samples from all organs of the polar bear were fixed in formalin, processed routinely for histopathological investigations, and stained with hematoxylin-eosin. Serum was not obtained, as the blood was severely hemolyzed. Additionally, liver sections were further stained with azan, Giemsa, Gordon-Sweet (reticulin), Hemalaun, von Kossa, periodic acid-Schiff, and Warthin-Starry.

### Transmission electron microscopy.

Native liver tissue and intestinal mucosa were homogenized and processed for negative staining using phosphotungstic acid and uranyl acetate for contrast. Samples of liver tissue fixed in a solution of 3.5% glutardialdehyde as well as liver tissue fixed in a solution of 4% formalin were processed for embedding in Epon 812 before preparing ultrathin sections and subsequent contrasting with uranyl acetate and lead citrate to search for viral particles by transmission electron microscopy (TEM).

### DNA/RNA extraction.

DNA and RNA were extracted from liver, prescapular lymph nodes, large intestine, kidney, and blood using Qiagen DNeasy Blood and Tissue kits according to the manufacturer’s protocol with the following modifications: tissue samples were lysed overnight for 15 h, and blood was lysed for 1 h. Samples were eluted in 150 µl. DNA was then quantified using an Agilent TapeStation (Agilent Technologies USA) using Genomic ScreenTapes and reagents.

### Generation of cDNA.

cDNA was generated from the DNA/RNA extract kit using the Invitrogen SuperScript IV (Thermo Fisher Scientific, USA) following the manufacturer’s instructions. Second-strand synthesis was then carried out by adding 1 µl of Klenow DNA polymerase I (Thermo Fisher Scientific, USA) to 21 µl of cDNA and incubated at 37°C for 60 min and then at 75°C for 20 min. The cDNA concentration was then determined with an Agilent TapeStation (Agilent Technologies, USA) using D1000 ScreenTapes and reagents.

### Illumina library preparation and sequencing.

The extracted DNA from tissue samples (liver, kidney, prescapular lymph node, and large intestine) were sheared to an average size of 350 bp using the Covaris M220. The fragmented DNA from each sample was subsequently used to generate Illumina libraries as previously described by Meyer and Kircher (32) with the modifications of Alfano et al. (33). The end of each library molecule was doubly indexed by assigning a unique set of P5 and P7 index adaptors (34) to prevent index jumping and to enable identification of pooled libraries after sequencing (35). The pooled libraries were amplified using Herculase II fusion polymerase (Agilent Technologies, USA) in 50-µl volumes with the following cycling conditions: (i) 95°C for 5 min; (ii) 8 cycles, with 1 cycle consisting of 95°C for 30 s, 60°C for 30 s, and 72°C for 40 s; and (iii) 72°C for 7 min. Each library was amplified with three replicates to minimize PCR bias on individual samples. The three replicates were then pooled and purified using the QIAquick PCR purification kit (Qiagen, Germany) and quantified with an Agilent TapeStation (Agilent Technologies USA) using D1000 ScreenTapes and reagents. The indexed amplified libraries were then pooled to equimolar amounts to a final concentration of 17.5 nM for paired-end sequencing on the Illumina NextSeq platform using the v2 reagent kit at the Berlin Center for Genomics in Biodiversity Research (BeGenDiv).

### Data analysis.

All fastq data generated were adaptor, size, and quality trimmed using Cutadapt v.1.5 (36), using a quality cutoff of 30 and minimum sequence length of 30 bp. To rule out contamination from other sources, each individual fastq file was mapped to the Ursus maritimus mitochondrial DNA (mtDNA) genome (NCBI accession no. AP012596) using Burrows-Wheeler Aligner (BWA-MEM, version 0.7.5a-r405) (37) to ensure that the mtDNA sequences obtained matched the species of interest. The resulting alignments were sorted using Samtools v 1.5 and visualize using Geneious v7.1 (38). Consensus sequences generated from the mtDNA mapping were subjected to a BLAST search in NCBI (39, 40) database for species confirmation.

### Viral Identification Pipeline.

Viral Identification Pipeline (VIP) is a recently developed bioinformatic pipeline that screens next-generation sequencing data for viral hits even when homology to known viruses is low. Each fastq file was processed using the VIP sense algorithm (41). The pipeline first aligns the fastq files to the Ursus maritimus reference genome and filters all reads that map to it. The remaining reads were further processed and filtered against a bacterial database. The reads remaining after this filtering step were further processed and searched against a nucleotide viral genome database followed by an amino acid alignment to viral protein database. The Geneious 7.1 (38) *de novo* assembler with medium sensitivity and Velvet version 1.2.10 (42) was then used to create contigs of the adenoviral positive reads (SRA accession no. PRJNA431169). Resulting contigs were aligned using MAFFT v. 7.017 (43) to create a genome consensus sequence. Consensus viral sequences were translated to all the available protein frames using Geneious 7.1 (38). The resulting reading frames were subjected to a BLAST search and used to annotate the viral genome (44).

### Phylogeny of novel adenovirus.

A multiple-sequence alignment was created using MAFFT v. 7.017 with adenoviral genome sequences obtained from GenBank (see Table S3 in the supplemental material) and the polar bear adenovirus 1 genome (GenBank accession no. MH115806) sequence. The amino acid sequences of *pol* and *hexon* gene-encoded proteins of PbAdV-1 (MH115806) were aligned using MAFFT v. 7.017 (43) with adenoviral sequences obtained from GenBank (Table S3). The resulting alignments were manually curated where needed. Jmodeltest2 (45) and Protest version 3.4 (46) were used to identify the appropriate sequence evolution model for the phylogenetic analysis. Phylogenetic trees were reconstructed using a maximum likelihood framework in the Hybrid build of RAxML (47). Nucleotide genome sequences were examined with the generalized time-reversible (GTR) substitution model with 20 maximum likelihood searches and 500 rapid bootstrap replicates. Amino acid sequences were examined with the Le-Gascuel (LG) gamma substitution model with 20 maximum likelihood searches and 500 rapid bootstrap replicates. For both nucleotide and amino acid phylogenies, frog adenovirus 1 (NC_002501.1) was used as an outgroup. Resulting trees were visualized using Geneious 7.1.

### Polar bear *CXADR* gene analysis.

The BWA-MEM algorithm (37) was used to align the whole-genome data obtained from Fritz to the data extracted from the polar bear genome *CXADR* gene (48). The resulting bam file was sorted, and a pileup was generated using SAMtools v1.5 (49). Nucleotide polymorphism screening was performed using BCFtools (50) and VCFtools (51).

### Adenovirus PCR of the *pol* and *hexon* genes.

PCR was used to confirm the adenovirus high-throughput sequencing (HTS) assemblies by using adenovirus-specific primers (Table 1) targeting the *pol* and *hexon* genes of the assembled viral genome. PCRs were run on DNA extracted from all tissue and blood samples and negative controls. The following PCR was used with the primers resulting in a 500- to 600-bp product: 12.5 µl of MyFi (Bioline USA), forward and reverse primers (10 mM), 4.5 µl of PCR-grade water, and 2 µl of DNA. The following thermocycling conditions were used: (i) 95°C for 5 min; (ii) 30 cycles, with 1 cycle consisting of 95°C for 20 s, 61°C for 20 s, and 72°C for 20 s; and (iii) 72°C for 2 min.

To rule out viral contamination, a significant problem in virome studies (17) from the DNA extraction process, a further PCR was carried out using MyTaq Blood-PCR kit, which directly amplifies PCR products from blood. The blood sample was diluted to 5% with PCR-grade water. Primers Hexon 1F (F stands for forward) and Hexon 1R (R stands for reverse) and Hexon 3F and Hexon 3R were used to amplify a 500- to 600-bp product according to the manufacturer’s protocol.

In addition, DNA was extracted as previously described from blood and liver samples from eight different polar bears collected from a range of zoos around Europe. The DNA from the eight samples was screened for the presence of the *hexon* and *pol* genes of the novel adenovirus using the PCR conditions described above (Table 2).

### qPCR for detection of a novel adenovirus.

To quantitatively determine the presence of the novel adenovirus SYBR green-based comparative *C*_*T*_ (ΔΔ*C*_*T*_) quantitative PCR (qPCR) was performed on 100 ng of adenovirus DNA from all tissue and blood samples. The qPCR targeted the *pol* gene of the novel adenovirus (GenBank accession no. MH115806). Primer concentrations were optimized according to the manufacturer’s protocol. The target genomic fragment (350 bp) was amplified using the following reaction mixture: 100 nM of the forward primer 5′ GGG GAG TGG GTC TAG AAA CT 3′ and reverse primer 5′ CGA AGA CTA TCA CGC CAA CA 3′, 12.5 µl SYBR Green JumpStart *Taq* ReadyMix (Sigma-Aldrich, USA), 0.25 µl reference dye (ROX), and 10 µl of template DNA. The PCR was carried out in strip tubes using the Stratagene MX3000P System (Agilent Technologies, USA) and StepOnePlus instruments under the following cycling conditions: 94°C for 2 min, followed by 40 cycles, with 1 cycle consisting of 94°C for 15 s and 58°C for 1 min. For all reactions, samples were run in either duplicate or triplicate along with positive and negative controls.

### Cell culture.

Crandell Rees feline kidney (CrFK), Madin-Darby canine kidney II (MDCK II), Madin-Darby bovine kidney (MDBK), African green monkey kidney (Vero), baby hamster kidney (BHK-21), rabbit kidney (RK-13), chicken fibroblast (DF1), human embryonic kidney (293T), and human cervical epithelial (HeLa) cells were propagated in Dulbecco’s modified Eagle’s medium (DMEM) (Biochrom) supplemented with 10% fetal bovine serum (FBS) (Biochrom), 100 U/ml penicillin, and 100 µg/ml streptomycin (1% penicillin-streptomycin). Equine dermal (ED) cells were grown in Iscove’s modified Dulbecco’s medium (IMDM, Pan) supplemented with 20% FBS, 1% nonessential amino acids (Biochrom), 1 mM sodium pyruvate (Pan), and 1% penicillin-streptomycin. Mouse neuroblastoma cells (N1e) were grown in Eagle’s minimum essential medium (MEM Eagle, Pan) supplemented with 5% FBS and 1% penicillin-streptomycin.

### Virus isolation.

To prepare inocula, tissue samples (prescapular lymph nodes, liver or kidney; 200 mg) were homogenized using a handheld microtube homogenizer, in which tissue in the microtube was triturated evenly using a plastic pestle by simple pushing in and pulling out the pestle in 1 ml of cold phosphate-buffered saline (PBS) with 2% penicillin and streptomycin and 5 µg/ml of amphotericin B (Biochrom) and incubated for 10 min at 4°C (52). Prepared tissue homogenates were frozen at −70°C and thawed to 4°C. Solid debris was pelleted by centrifugation at 7,000 rpm for 10 min at 4°C. Clarified homogenates were used as an inoculum, applied over different cell lines, incubated at 37°C, and observed daily for the development of cytopathic effect (CPE). Each inoculated cell line was subjected to three blind passages.

### Indirect immunofluorescence assay.

An indirect immunofluorescence assay (IFA) was performed to detect adenoviral antigen in cell culture. CrFK, MDCK II, and Vero cells were grown in 24-well plates and inoculated with 200 µl of the virus sample. After 48 h, cells were washed twice with PBS, fixed with 4% paraformaldehyde for 30 min, and permeabilized with 0.1% Triton X-100 for 10 min. Following each step, plates were washed with PBS. Permeabilized cells were blocked with 3% bovine serum albumin (BSA) (VWR Life Sciences) in PBS for 1 h at room temperature and incubated with goat antiadenovirus primary antibody (catalog no. 0151-9004; Bio-Rad) diluted 1:20 in blocking buffer at 4°C overnight. Further, fluorescein isothiocyanate (FITC)-labeled rabbit anti-goat IgG H&L (ab6737; Abcam) secondary antibody diluted to 1:100 in blocking buffer was applied and incubated for 1 h at room temperature. Mock-infected CrFK cells were stained with primary and secondary antibodies of the same dilution. Plates were analyzed using a Zeiss Axio Vert.A1 fluorescence microscope.

10.1128/mSphere.00171-18.6 TABLE S4  Nucleotide percentage pairwise identity of polar bear *CXADR* gene compared to other *CXADR* genes found in mammals. Download TABLE S4, DOCX file, 0.01 MB. Copyright © 2018 Dayaram et al. 2018 Dayaram et al. This content is distributed under the terms of the Creative Commons Attribution 4.0 International license.

### Data availability.

Contigs of the adenoviral positive reads from the Illumina data are available in the NCBI Sequence Read Achieve (SRA) under accession no. PRJNA431169. The PbAdV-1 genome is available in Genbank under accession no. MH115806.
