# Supplementary figures and images for: Novel Divergent Polar Bear-Associated Mastadenovirus Recovered from a Deceased Juvenile Polar Bear
Source: mSphere. 2018 Jul 25;3(4):e00171-18. doi: 10.1128/mSphere.00171-18 (PMC6060345; doi:10.1128/mSphere.00171-18)

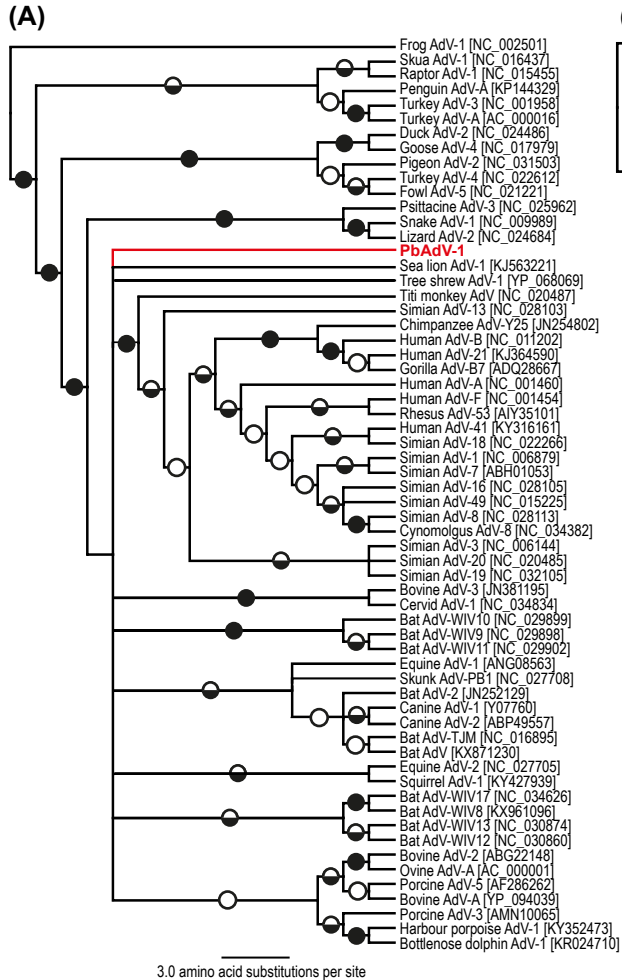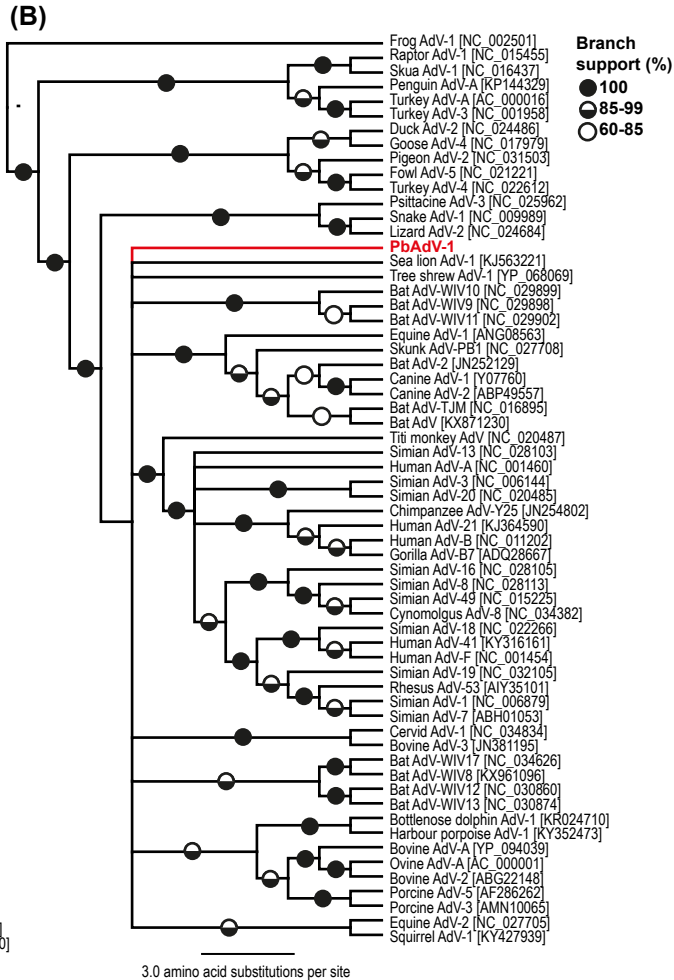

Supplement: FIG S1 [file sph004182597sf1.pdf]

## Positive samples

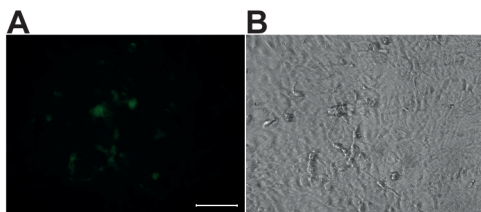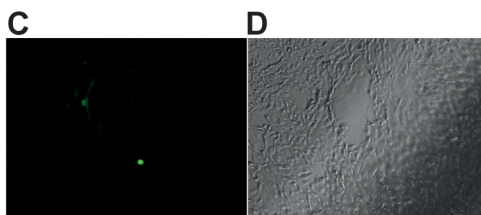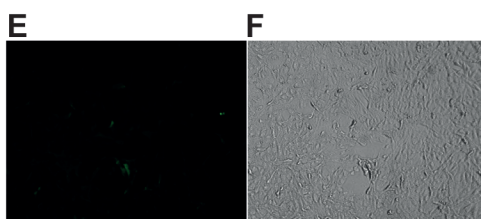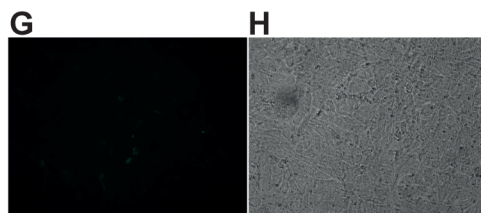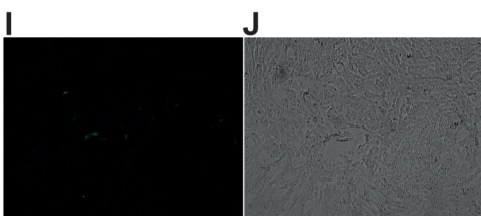

## Negative control samples

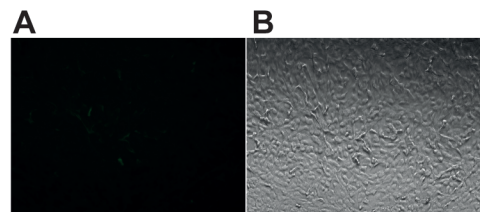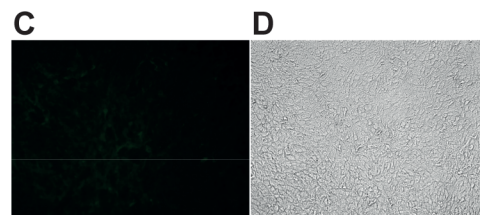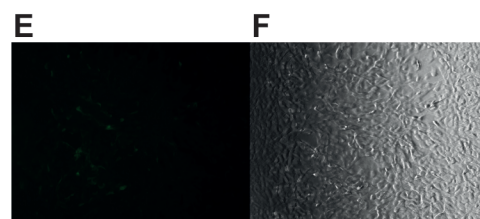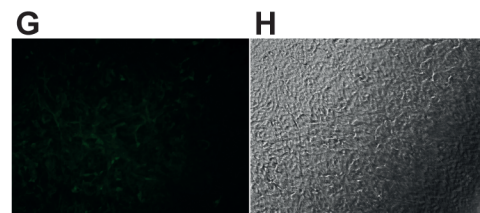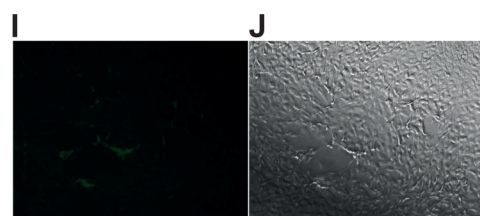

Supplement: FIG S2 [file sph004182597sf2.pdf]
