# Supplementary material for: Novel Divergent Polar Bear-Associated Mastadenovirus Recovered from a Deceased Juvenile Polar Bear
Source: mSphere. 2018 Jul 25;3(4):e00171-18. doi: 10.1128/mSphere.00171-18 (PMC6060345; doi:10.1128/mSphere.00171-18)
Supplement: TABLE S1 [file sph004182597st1.docx]

| **Primer name** | **Primer 5' to 3'** |
| --- | --- |
| Hexon 1F | GTC CGA ACA GAC CGA ACT AC |
| Hexon 1R | TCC TCT TAA GGC TAT CCG GG |
| Hexon 2F | ATT AAT CTG GCG GCG AAC AT |
| Hexon 2R | TCC GAG AAG GGA TGG AGA TC |
| Hexon 3F | GAC TCT TCT GTT AGC TGG CC |
| Hexon 3R | CGC CCA TGG ACA TGA AGT TA |
| pol 1F | CAT GAC CTT TAG CTC GCA GT |
| pol 1R | TGC TTC CTT TGT TCT CGC TT |
| pol 2F | CTG ACA TGA AGG CTC TGG TC |
| pol 2R | CCC CTT TCT GTT CCC GAA AA |
| pol 3F | GGG GAG TGG GTC TAG AAA CT |
| pol 3R | CGA AGA CTA TCA CGC CAA CA |
