# Supplementary material for: Novel Divergent Polar Bear-Associated Mastadenovirus Recovered from a Deceased Juvenile Polar Bear
Source: mSphere. 2018 Jul 25;3(4):e00171-18. doi: 10.1128/mSphere.00171-18 (PMC6060345; doi:10.1128/mSphere.00171-18)
Supplement: TABLE S2 [file sph004182597st2.docx]

| **Polar bear** | **Origin of sample** | **Sample type** | **Detection by PCR** | | | | | |
| --- | --- | --- | --- | --- | --- | --- | --- | --- |
|  |  |  | **Hexon 1** | **Hexon 2** | **Hexon 3** | **pol 1** | **pol 2** | **pol 3** |
| Knut | Berlin | Blood | - | - | - | - | - | - |
| Lars | Copenhagen | Blood | - | - | - | - | - | - |
| Anton | Stuttgart | Blood | - | - | - | - | - | - |
| Anastasia | Berlin | Blood | - | - | - | - | - | - |
| Tosca | Berlin | Blood | - | - | - | - | - | - |
| Troll | Norway | Blood | - | - | - | - | - | - |
| Nancy | Berlin | Liver | - | - | - | - | - | - |
| Wilma | Nuremberg | Liver | - | - | - | - | - | - |

-/+ denotes a negative or positive result from PCR
