# Supplementary material for: Novel Divergent Polar Bear-Associated Mastadenovirus Recovered from a Deceased Juvenile Polar Bear
Source: mSphere. 2018 Jul 25;3(4):e00171-18. doi: 10.1128/mSphere.00171-18 (PMC6060345; doi:10.1128/mSphere.00171-18)
Supplement: TABLE S3 [file sph004182597st3.docx]

| **Adenovirus sequence** | **Species origin** | **Scientific name** | **GenBank Accession No.** |
| --- | --- | --- | --- |
| Bat AdV | Bat | *Corynorhinus rafinesquii* | KX871230 |
| Bat AdV-2 | Bat | *Pipistrellus pipistrellus* | JN252129 |
| Bat AdV-TJM | Bat | *Myotis ricketti* | NC_016895 |
| Bat AdV-WIV10 | Bat | *Rhinolophus sinicus* | NC_029899 |
| Bat AdV-WIV11 | Bat | *Rhinolophus sinicus* | NC_029902 |
| Bat AdV-WIV12 | Bat | *Miniopterus schreibersi* | NC_030860 |
| Bat AdV-WIV13 | Bat | *Miniopterus schreibersi* | NC_030874 |
| Bat AdV-WIV17 | Bat | *Rousettus leschenaultii* | NC_034626 |
| Bat AdV-WIV8 | Bat | *Rousettus leschenaultii* | KX961096 |
| Bat AdV-WIV9 | Bat | *Rhinolophus sinicus* | NC_029898 |
| Bottlenose dolphin AdV-1 | Bottlenose dolphin | *Tursiops truncatus* | KR024710 |
| Bovine AdV-2 | Cow | Bovine | ABG22148 |
| Bovine AdV-3 | Cow | Bovine | JN381195 |
| Bovine AdV-A | Cow | Bovine | YP_094039 |
| Canine AdV-1 | Dog | Canine | Y07760 |
| Canine AdV-2 | Dog | Canine | ABP49557 |
| Cervid AdV-1 | White-tailed deer | *Odocoileus virginianus* | NC_034834 |
| Chimpanzee AdV-Y25 | Chimpanzee | *Pan troglodytes* | JN254802 |
| Cynomolgus AdV-8 | Cynomolgus monkey | *Macaca fascicularis* | NC_034382 |
| Duck AdV-2 | Muscovy duck | *Cairina moschata* | NC_024486 |
| Equine AdV-1 | Horse | *Equus caballus* | KU133477 |
| Equine AdV-2 | Horse | *Equus caballus* | NC_027705 |
| Fowl AdV-5 | Chicken | *Gallus gallus* | NC_021221 |
| Frog AdV-1 | Frog | Anura | NC_002501 |
| Goose AdV-4 | Goose | *Anser domestica* | NC_017979 |
| Gorilla AdV-B7 | Gorilla | *Gorilla g. gorilla* | ADQ28667 |
| Harbour porpoise AdV-1 | Harbour porpoise | *Phocoena phocoena* | KY352473 |
| Human AdV-21 | Human | *Homo sapien* | KJ364590 |
| Human AdV-41 | Human | *Homo sapien* | KY316161 |
| Human AdV-A | Human | *Homo sapien* | NC_001460 |
| Human AdV-B | Human | *Homo sapien* | NC_011202 |
| Human AdV-F | Human | *Homo sapien* | NC_001454 |
| Lizard AdV-2 | Mexican beaded lizard | *Heloderma horridum* | NC_024684 |
| Ovine AdV-A | Sheep | *Ovis aries* | AC_000001 |
| Penguin AdV-A | Chinstrap penguin | *Pygoscelis antarcticus* | KP144329 |
| Pigeon AdV-2 | Domestic pigeon | *Columba livia* | NC_031503 |
| Porcine AdV-3 | Pig | *Sus scrofa* | AMN10065 |
| Porcine AdV-5 | Pig | *Sus scrofa* | AF286262 |
| Psittacine AdV-3 | Southern mealy parrot | *Amazona farinosa* | NC_025962 |
| Raptor AdV-1 | Harris hawk | *Parabuteo unicinctus* | NC_015455 |
| Rhesus AdV-53 | Rhesus macaque | *Macaca mulatta* | AIY35101 |
| Sea lion AdV-1 | California sea lion | *Zalophus californianus* | KJ563221 |
| Simian AdV-1 | Cynomolgus monkey | *Macaca fascicularis* | NC_006879 |
| Simian AdV-13 | Simian | *Macaca sp.* | NC_028103 |
| Simian AdV-16 | Grivet | *Cercopithecus aethipos* | NC_028105 |
| Simian AdV-18 | Grivet | *Cercopithecus aethipos* | NC_022266 |
| Simian AdV-19 | Yellow baboon | *Papio cynocephalus* | NC_032105 |
| Simian AdV-20 | Grivet | *Cercopithecus aethipos* | NC_020485 |
| Simian AdV-3 | Rhesus macaque | *Macaca mulatta* | NC_006144 |
| Simian AdV-49 | Cynomolgus monkey | *Macaca fascicularis* | NC_015225 |
| Simian AdV-7 | Rhesus macaque | *Macaca mulatta* | ABH01053 |
| Simian AdV-8 | Cynomolgus monkey | *Macaca fascicularis* | NC_028113 |
| Skua AdV-1 | South Polar Skua | *Stercorarius maccormicki* | NC_016437 |
| Skunk AdV-PB1 | North American skunk | *Conepatus leuconotus* | NC_027708 |
| Snake AdV-1 | Corn snake | *Elaphe guttata* | NC_009989 |
| Squirrel AdV-1 | Red squirrel | *Sciurus vulgaris* | KY427939 |
| Titi monkey AdV | Titi monkey | *Callicebus cupreus* | NC_020487 |
| Tree shrew AdV-1 | Tree shrew | *Tupaia belangeri* | YP_068069 |
| Turkey AdV-3 | Turkey | Meleagris | NC_001958 |
| Turkey AdV-4 | Turkey | *Meleagris gallopavo* | NC_022612 |
| Turkey AdV-A | Turkey | Meleagris | AC_000016 |
