# Supplementary material for: Novel Divergent Polar Bear-Associated Mastadenovirus Recovered from a Deceased Juvenile Polar Bear
Source: mSphere. 2018 Jul 25;3(4):e00171-18. doi: 10.1128/mSphere.00171-18 (PMC6060345; doi:10.1128/mSphere.00171-18)
Supplement: TABLE S4 [file sph004182597st4.docx]

| **Species** | **GenBank accession no.** | **% identity to polar bear CXADR gene** |
| --- | --- | --- |
| Giant panda | NW_003217340 | 83.59 |
| Hawaiian monk seal | NW_018734271 | 82.03 |
| Amur tiger | NW_006711831 | 65.16 |
| Domestic cat | NC_018731 | 61.52 |
| Ass | NW_014638543 | 48.18 |
| Horse | NC_009169 | 48.48 |
| Przewalski’s horse | NW_007676649 | 48.40 |
| Flying fox | NW_011888819 | 48.88 |
| Black flying fox | NW_006433378 | 46.71 |
| Dog | NC_006613 | 54.37 |
| Bottlenose dolphin | NW_017844625 | 43.76 |
